# Relationship between the Controlling Nutritional Status Score and Infrainguinal Bypass Surgery Outcomes in Patients with Chronic Limb-threatening Ischemia

**DOI:** 10.3400/avd.oa.21-00132

**Published:** 2021-12-25

**Authors:** Satoshi Yamamoto, Juno Deguchi, Takuya Hashimoto, Masamitsu Suhara, Osamu Sato

**Affiliations:** 1 Department of Vascular Surgery, Saitama Medical Center, Saitama Medical University, Kawagoe, Saitama, Japan

**Keywords:** peripheral arterial disease, chronic limb-threatening ischemia, Controlling Nutritional Status Score, survival, limb salvage

## Abstract

**Objective:** We investigated the association between Controlling Nutritional Status (CONUT) scores and the outcomes of bypass surgery in patients with chronic limb-threatening ischemia (CLTI).

**Methods:** We retrospectively calculated preoperative CONUT scores in 118 patients (127 limbs) with CLTI who underwent infrainguinal bypass surgery. Survival, graft patency, and limb salvage were compared between the high and low CONUT score groups based on the respective cutoff points.

**Results:** The median and mean CONUT scores were 5 and 4.8, respectively. The postoperative survival rate was lower in the high CONUT score (3–12) group than in the low CONUT score (0–2) group (P=0.0043). The limb salvage rate after arterial reconstruction was also significantly lower in the high CONUT score (8–12) group than in the low CONUT score (0–7) group (P=0.0009).

**Conclusions:** The CONUT score can predict infrainguinal bypass surgery outcomes in patients with CLTI. (This is a translation of J Jpn Coll Angiol 2020; 60: 35–41.)

## Introduction

The prevalence of multiple diseases such as diabetes mellitus, ischemic heart disease, and renal failure is high among patients with chronic limb-threatening ischemia (CLTI), and their prognosis is usually poor. Patients with critical limb ischemia [The trans-Atlantic Inter-Society Consensus II (TASC II) grade] have a death rate of approximately 20% at 1 year after onset; therefore, their prognosis is worse than that of patients with malignant diseases.^1,2)^ In the treatment of CLTI, it is necessary to consider the life expectancy of patients and determine the best treatment enabling limb salvage for each patient. Currently, although risk factors for death and major amputation of limbs have been reported, predictive indicators for life expectancy and limb salvage have not been established. Based on the Bypass versus Angioplasty in Severe Ischemia of the Leg (BASIL-2) trial, surgical bypass is recommended as the primary revascularization strategy when there is a suitable autologous vein graft and the patient’s life expectancy is at least 2 years.^3–5)^ However, it is difficult to predict whether patients will survive for more than 2 years, and the selection criteria for surgical bypass or endovascular treatment are practically undefined. Therefore, in clinical practice, surgical bypass is often avoided based on subjective judgment, and endovascular treatment, which can often be ineffective, is frequently and conveniently selected as the first choice of treatment. In contrast, although surgical bypass is performed actively and may lead to limb salvage, death sometimes occurs before the ulcer or necrosis has healed. When considering revascularization surgery in the treatment of CLTI, objective predictive indicators of life expectancy and limb salvage are desirable.

In patients with CLTI, a single factor is unlikely to predict expectancy due to the diversity of comorbid diseases and the complexity of the pathological condition. For example, the serum levels of proteins, such as albumin, fluctuate highly due to inflammation, hepatic and renal functions, dehydration, and fluid infusion. However, a scoring system that combines multiple items can be useful in selecting a treatment strategy through comprehensive risk stratification. Multi-item nutritional and immunological evaluation indices have been reported to be useful as predictive indicators of mortality and related events.^6,7)^ The Controlling Nutritional Status (CONUT) score, a multi-item nutritional evaluation measure, has been suggested to be useful as a factor predicting life prognosis in patients with gastrointestinal cancer and heart failure.^8–13)^ However, it is unclear whether the CONUT score is useful in determining the expectancy of patients with CLTI after they have undergone surgical bypass surgery.

When infrainguinal revascularization is required for CLTI treatment, it is necessary to consider surgical bypass even if other treatment options are chosen. In this study, we investigated the association between the CONUT score and treatment outcomes in patients with CLTI who underwent infrainguinal surgical bypass in our department.

## Subjects and Methods

From 2008 to 2018, 127 limbs of 118 patients who underwent infrainguinal bypass surgery for CLTI with tissue loss due to atherosclerosis obliterans and whose CONUT scores could be calculated were included in the study. The CONUT score was calculated as the sum of scores for preoperative serum albumin level, total lymphocyte count, and total cholesterol level (**Table 1**). The risk group categorization for nutritional status based on the CONUT score was as follows: 0–1=normal, 2–4=mild, 5–8=moderate, and 9–12=severe.^8)^ The values of each measurement were the most recent values measured before the revascularization procedure was performed (in principle, within 1 week for serum albumin level and total lymphocyte count and within approximately 1 month for total cholesterol level).

**Table 1:** Table 1 CONUT Score

Parameter				
Serum albumin (g/dL)	3.5–4.5	3.0–3.49	2.5–2.9	<2.5
Albumin score	0	2	4	6
Total lymphocytes (count/mL)	>1600	1200–1599	800–1199	<800
Lymphocyte score	0	1	2	3
Total cholesterol (mg/dL)	>180	140–180	100–139	<100
Cholesterol score	0	1	2	3
Screening total score (CONUT score)	Albumin score+Lymphocyte score+Cholesterol score			
Undernutrition degree	Normal	Light	Moderate	Severe
CONUT score	0–1	2–4	5–8	9–12

CONUT: Controlling Nutritional Status

As a surgical strategy, if the patient was judged to be operable as per preoperative systemic evaluation, infrainguinal revascularization was actively performed for the purpose of limb salvage. Preoperative contrast-enhanced computed tomography (CT), angiography, and ultrasonography were performed, and in principle, bypass surgery was considered to ensure in-line flow to the foot. Patients without an appropriate arterial anastomosis or autologous vein graft for distal bypass were considered ineligible for limb bypass or subjected to incomplete revascularization and were not included in this study. In addition, patients with necrosis or infection extending above the heel were not included in this study because they were not eligible for limb salvage bypass surgery. In the postoperative period, graft patency was confirmed by palpation, Doppler auscultation, ultrasonography (duplex scan), or CT scan at 1 week, 1 month, 3 and 6 months, 1 year, and every 6 months–1 year thereafter. Additionally, the presence or absence of surgical wound complications (wound infection and dehiscence), excluding ulcers and necrotic areas on the foot, was examined.

The relationship between the preoperative CONUT score and patient background, clinical findings, survival rate, limb salvage rate, and surgical wound complications obtained in this study was retrospectively evaluated. For statistical analysis, Student’s t-test or Chi-square/Fisher’s exact test was used. Patients with survival of >1 year, limb salvage, and graft patency were considered as discriminant groups, and the cut-off value of the CONUT score was calculated by the Youden Index and the receiver operating characteristic curve. The survival, limb salvage, and graft patency rates were compared. For constructing limb salvage, survival, and graft patency curves, Kaplan–Meier method was used, and the difference between the two groups was determined using the log-rank test. The threshold for statistical significance was set at P<0.05.

This study was conducted in accordance with the ethical guidelines for medical research involving human subjects and approved by the Institutional Review Board of the Saitama Medical Center (approval no.: 2008).

## Results

**Figure 1:**
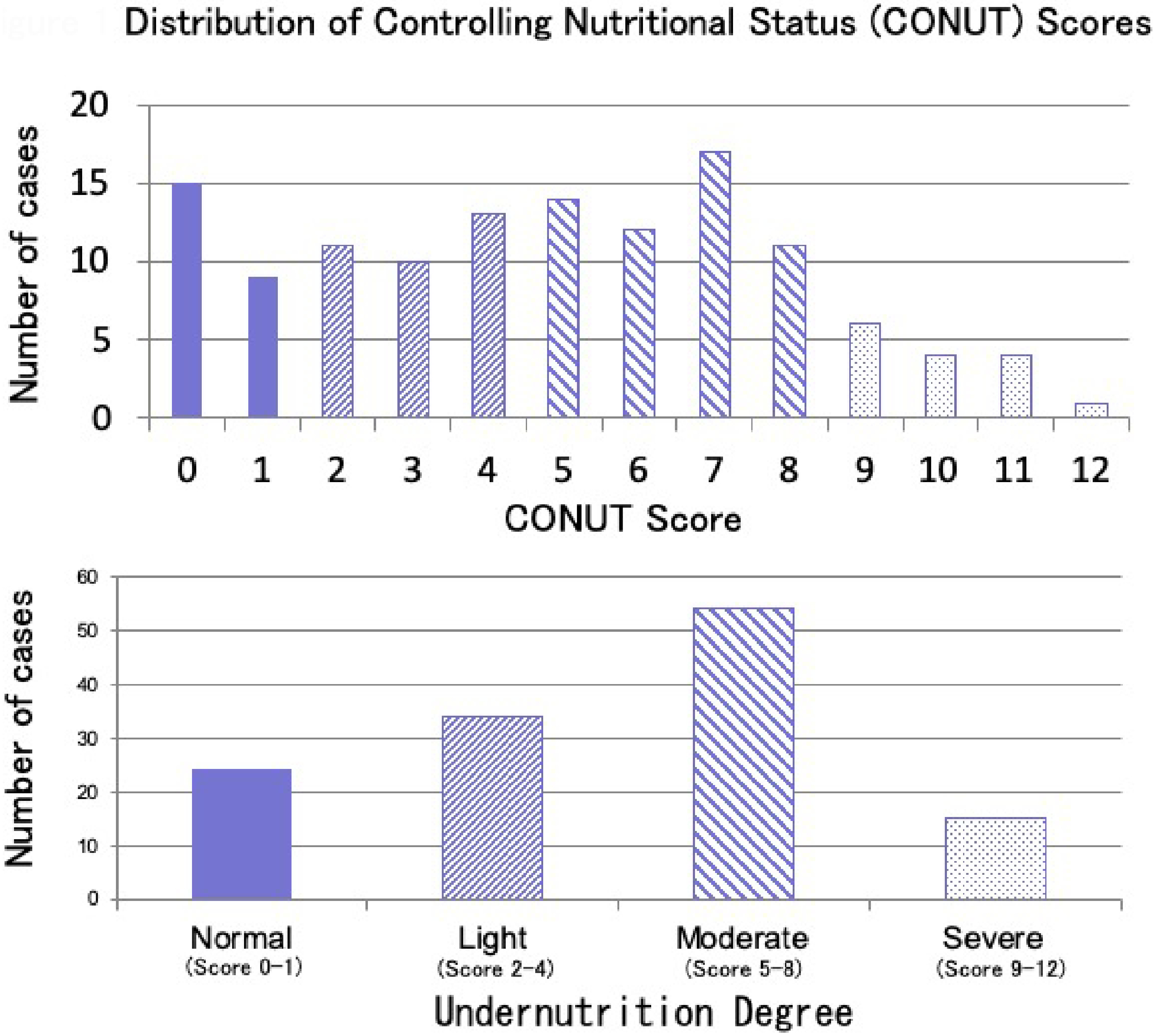
Fig. 1 The median and mean Controlling Nutritional Status Scores were 5 and 4.8, respectively.

The patient backgrounds are shown in **Table 2**. As per total cholesterol levels, there were 44 patients with coexisting dyslipidemia; however, there was no significant difference between these and other patients (167±48 mg/dL for patients with coexisting dyslipidemia and 165±43 mg/dL for patients without coexisting dyslipidemia, P=0.90). Thirty-seven patients were using statins; however, their total cholesterol levels did not differ significantly from those of patients not using statins (160±50 mg/dL for statin users and 168±43 mg/dL for non-statin users, P=0.37). The distribution of the preoperative CONUT score is shown in **Fig. 1** (median: 5 points, mean: 4.8 points), and the most frequent score was 7 points. For comparison, patients with CONUT scores of 0–5 were classified into the lower score group (n=72) and those with CONUT scores of 6–12 into the higher score group (n=55). The higher score group had more males, more cases of hypertension and diabetes mellitus, and significantly more cases of end-stage renal failure (requiring dialysis) (**Table 2**) compared to the lower score group. With regard to the cause of death, cardiovascular events and sepsis (infection) were common (**Table 3**). However, in the lower score group, cardiovascular events and sepsis together accounted for 70% of deaths, whereas in the higher score group, they accounted for only 40% of deaths. Other causes of death, such as digestive diseases, were diverse.

**Table 2:** Table 2 Baseline clinical characteristics based on the CONUT Score

	All (n=127)	Lower score group (CONUT score: 0–5) (n=72)	Higher score group (CONUT score 6–12) (n=55)	P value
Age (years), mean±SD	72.5±8.9	71.8±9.4	73.4±8.3	0.30
Male (%)	94 (74%)	48 (67%)	46 (84%)	0.041
Smoking history, n (%)	91 (72%)	48 (67%)	43 (78%)	0.17
Comorbid diseases				
Hypertension, n (%)	105 (83%)	55 (76%)	50 (91%)	0.036
Dyslipidemia, n (%)	44 (35%)	28 (39%)	16 (29%)	0.27
Diabetes mellitus, n (%)	103 (81%)	53 (74%)	50 (91%)	0.021
Coronary artery disease, n (%)	44 (35%)	25 (35%)	19 (35%)	1.00
Chronic heart failure, n (%)	21 (17%)	9 (13%)	12 (22%)	0.23
Cerebrovascular disease, n (%)	25 (20%)	12 (17%)	13 (24%)	0.37
ESRD on hemodialysis, n (%)	56 (44%)	21 (29%)	35 (64%)	0.0001
Medications				
Warfarin, n (%)	25 (20%)	12 (17%)	13 (20%)	0.37
Aspirin, n (%)	59 (46%)	32 (44%)	27 (49%)	0.72
Thienopyridine derivatives, n (%)	28 (22%)	15 (21%)	13 (24%)	0.83
Statins, n (%)	37 (29%)	21 (29%)	16 (29%)	1.00
Laboratory data				
Serum albumin (g/dL), mean±SD	3.13±0.59	3.51±0.40	2.63±0.38	<0.0001
White blood cells (count/µL), mean±SD	8843±3615	7906±2131	10071±4670	0.0021
Lymphocytes (count/µL), mean±SD	1307±617	1532±606	1012±500	<0.0001
Total cholesterol (mg/dL), mean±SD	166±45	187±44	138±27	<0.0001
Distal anastomosis site in infrainguinal arterial reconstruction				
Popliteal artery above knee, n (%)	17 (13%)	11 (15%)	6 (11%)	—
Popliteal artery below knee, n (%)	20 (16%)	10 (14%)	10 (18%)	—
Tibioperoneal trunk, n (%)	3 (2%)	3 (4%)	0 (0%)	—
Anterior tibial artery, n (%)	28 (22%)	15 (21%)	13 (24%)	—
Posterior tibial artery, n (%)	24 (19%)	14 (19%)	10 (18%)	—
Peroneal artery, n (%)	9 (7%)	6 (8%)	3 (5%)	—
Dorsal pedis artery, n (%)	19 (15%)	9 (13%)	10 (18%)	—
Plantar artery, n (%)	7 (6%)	4 (6%)	3 (5%)	—

CONUT: Controlling Nutritional Status; ESRD: end-stage renal disease

**Table 3:** Table 3 Leading causes of death

Leading causes of death	All (n=34)	Lower score group (score 0–5) (n=17)	Higher score group (score 6–12) (n=17)
Cardiovascular event, n (%)	11 (32%)	7 (41%)	4 (24%)
Heart failure	8	4	4
Myocardial infarction	1	1	0
Arrhythmia (VT/VF)	2	2	0
Cerebrovascular event, n (%)	2 (6%)	1 (6%)	1 (6%)
Bleeding	1	0	1
Infarction	1	1	0
Pneumonia, n (%)	2 (6%)	1 (6%)	1 (6%)
Renal failure, n (%)	2 (6%)	1 (6%)	1 (6%)
Gastrointestinal event, n (%)	2 (6%)	0 (0%)	2 (12%)
Bleeding	1	0	1
Peritonitis	1	0	1
Pancreatitis/cholangitis, n (%)	1 (3%)	0 (0%)	1 (6%)
Malignant Disease^a^, n (%)	1 (3%)	0 (0%)	1 (6%)
Sepsis, n (%)	8 (24%)	5 (29%)	3 (18%)
Suicide, n (%)	1 (3%)	0 (0%)	1 (6%)
Unknown, n (%)	4 (12%)	2 (12%)	2 (12%)

VT: ventricular tachycardia; VF: ventricular fibrillation ^a^ Gastric cancer

**Figure 2:**
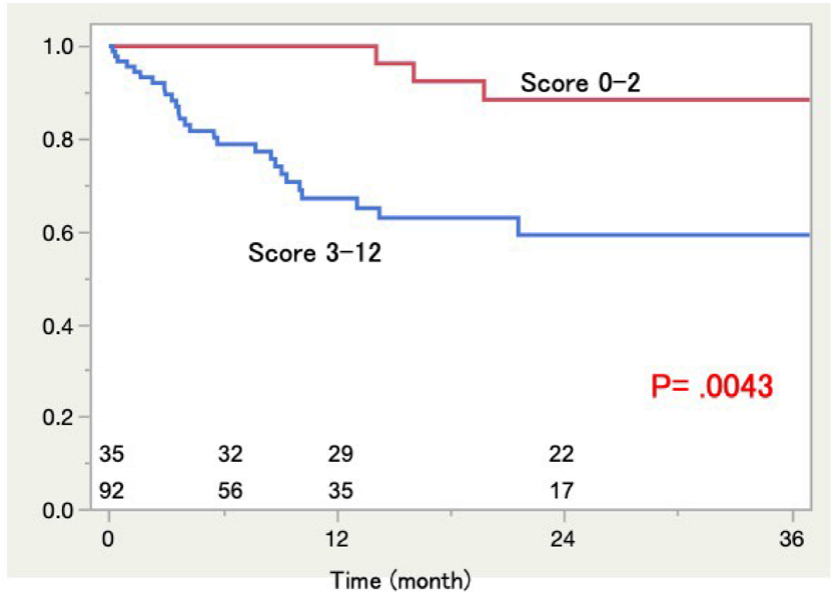
Fig. 2 Survival rates of the patients with higher Controlling Nutritional Status (CONUT) Scores (3–12) and those with lower CONUT Scores (0–2) after arterial reconstruction. The postoperative survival rate was lower in the higher score group than in the lower score group (P=0.0043).

For survival rate, a score of 2 [sensitivity 45%, specificity 100%, area under the curve (AUC) 0.764] was calculated as the cut-off value, and the survival rate of high CONUT score group (3–12 points) was significantly lower than that of the low CONUT score group (0–2 points) (one-year survival rate: 67% vs. 100%, P=0.0043) (**Fig. 2**).

**Figure 3:**
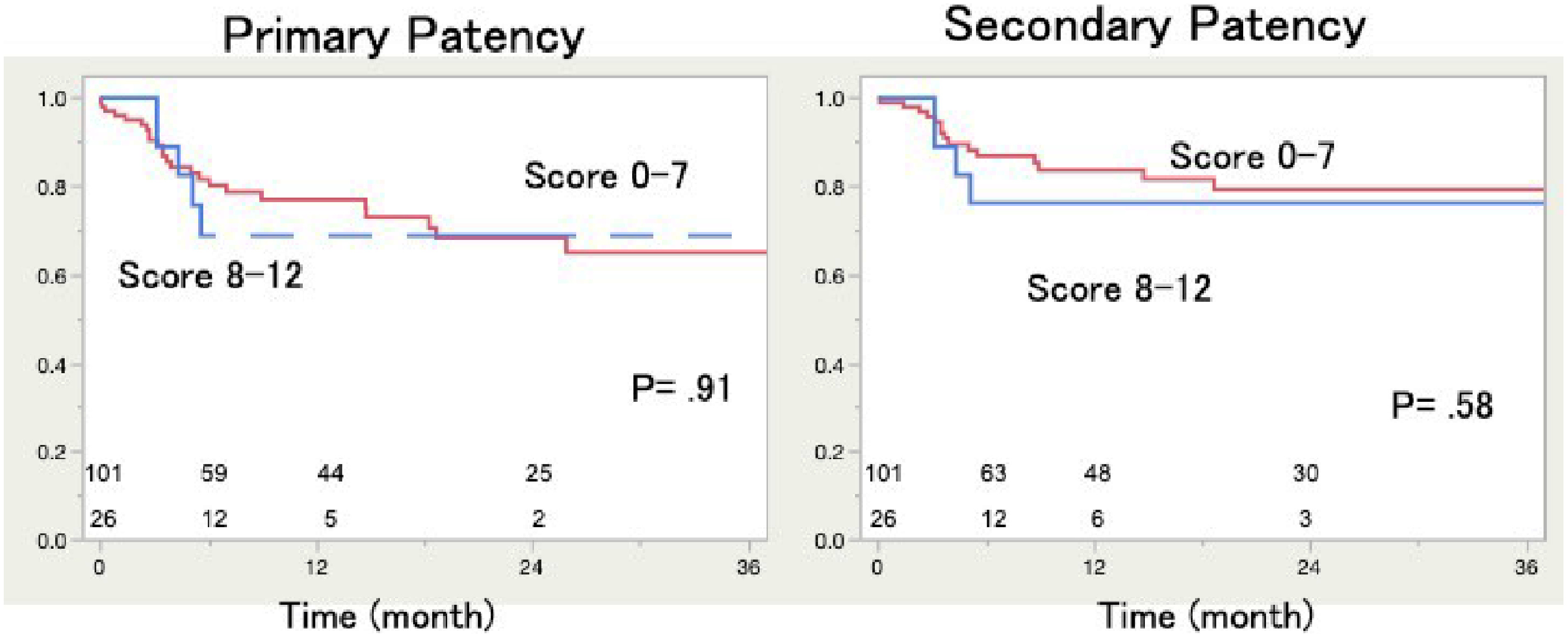
Fig. 3 Graft patency rates of patients with higher Controlling Nutritional Status (CONUT) scores (8–12) and those with lower CONUT scores (0–7). There was no signiﬁcant diﬀerence in primary and secondary graft patency between the higher and the lower score groups (p=0.91 and 0.58, respectively).

**Figure 4:**
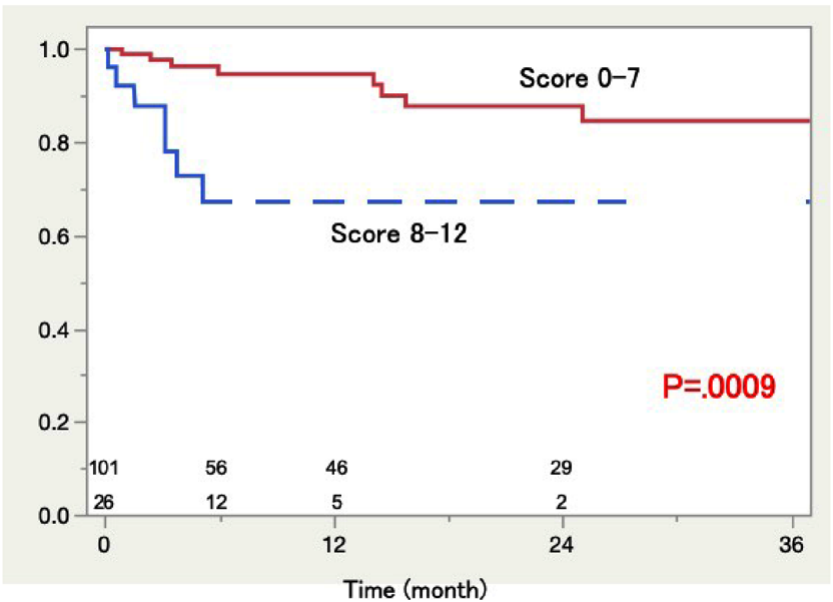
Fig. 4 Limb salvage in patients with higher Controlling Nutritional Status (CONUT) scores (8–12) and those with lower CONUT scores (0–7) after arterial reconstruction. The overall limb salvage rate was lower in the higher score group than in the lower score group (P=0.0009).

A score of 7 (sensitivity 91%, specificity 21%, AUC 0.501) was considered as the cut-off value for graft patency (primary) rate, whereas a score of 7 (sensitivity 92%, specificity 64%, AUC 0.865) was considered as the cut-off value for limb salvage rate. Graft patency rate did not differ significantly between the two groups for both primary and secondary patencies, regardless of the cut-off value (**Fig. 3**); however, the limb salvage rate was significantly lower in the high CONUT score group (8–12 points) than in the low CONUT score group (0–7 points) (one-year limb salvage rate 67% vs. 95%, P=0.0009) (**Fig. 4**). On the other hand, there was no significant difference in the limb salvage rate between the dialysis and non-dialysis groups (P=0.28).

Among 127 limbs included in the study, surgical wound complications were observed in 27 limbs [wound infection: 7 limbs (6%) and wound dehiscence: 20 limbs (16%)]. From the point of view of surgical wound complications, a score of 9 (sensitivity 19%, specificity 90%, AUC 0.512) was considered as the cut-off value; and no significant difference was observed between the two groups divided as per the cut-off value (P=0.39).

## Discussion

Our department has been actively performing bypass surgery for the purpose of limb salvage in patients with CLTI. This study suggests that the CONUT score, an index of a patient’s nutritional and immunological statuses, may be one of the indicators for life expectancy after infrainguinal bypass surgery in patients with CLTI. This result is consistent with the fact that the CONUT score is a prognosis indicator in various diseases such as gastrointestinal cancer and heart failure. On the other hand, it is known that the life prognosis of patients undergoing dialysis is poor.^4,5)^ A high CONUT score is common in dialysis patients, and the CONUT score is considered to be associated with dialysis. Therefore, it is necessary to further investigate the potential of the CONUT score as a life expectancy-predicting factor separately in the dialysis and non-dialysis patient groups. In this study, the CONUT score was suggested to be a life expectancy-predicting factor, especially in non-dialysis patients; however, the number of patients in this study was not sufficient to provide a certain opinion on the subgroup analysis results. Thus, we would like to increase the number of patients in further studies. Furthermore, the CONUT score is an overall risk scoring system that combines the risk of each organ disease and also stratifies the risk of cases with poor nutritional and immunological statuses other than dialysis cases although it is associated with dialysis. In this study, the causes of death in the high CONUT score group (besides cardiovascular events and sepsis) were diverse; therefore, the CONUT score can be used as a reference for predicting overall life expectancy.

The CONUT score in this study was generally high, with a median of 5 points. When referring to the CONUT score of patients with other diseases in our department, the median score was 2 points (mean 2.0 points) for abdominal aortic and iliac artery aneurysm surgery cases (53 cases in 2018). The mean score was less than 3 in cases of gastrointestinal cancer and heart failure in many reports.^9–13)^ Compared with other disease groups, the CLTI surgery group had the worst nutritional and immunological statuses. Since the CONUT score was not originally developed for patients with CLTI, in this study, the risk group classification was used as a reference, and the low (0–5 points) and high (6–12 points) CONUT score groups were compared in terms of patient backgrounds and causes of death. In addition, since the cut-off values differed depending on the purpose of evaluation, the cut-off values for survival, limb salvage, graft patency, and wound complications were calculated for each group separately before comparison. The CONUT score should be examined for each disease and each evaluation purpose.

The CONUT score is not only associated with survival but also with perioperative complications in patients with gastrointestinal cancer and cardiovascular diseases and length of hospital stay in patients with heart failure.^9–13)^ In the present study, in addition to survival, differences in limb salvage rates were also observed. This may be because the CONUT score, which reflects the nutritional and immunological statuses, was associated with delayed healing and infection of ulcers and necrotic areas related to limb amputation. In this study, we found no significant difference in the limb salvage rate between dialysis and non-dialysis patients; therefore, the CONUT score may be a useful predictive indicator of limb prognosis in patients undergoing CLTI surgery. In the present study, there was no direct relationship between the CONUT score and surgical wound complications, and the healing time of ulcers and necrotic areas could not be evaluated due to the limited availability of information. However, the CONUT score may correlate with the healing time of ulcers and necrotic areas, which requires further investigation. If the healing time of ulcers and necrosis is expected to be long, primary major amputation may be an option for patients who are not expected to have high life expectancy.

In actual clinical practice, a scoring system consisting of subjective items and specific tests is difficult to use. In this respect, the CONUT score is a simple and easy-to-use screening test because all items are objective indicators based on general blood tests. The total lymphocyte count is considered to reflect the immunological capacity, and since the immunological capacity is decreased in a low-nutritional state, there is a correlation between the nutritional status and immunological capacity.^14)^ In this study, in patients who underwent multiple blood sampling tests before surgery, the total lymphocyte counts fluctuated a little (although the neutrophil count fluctuated greatly) and the percentage of lymphocytes fluctuated due to inflammatory findings. This suggests that the total lymphocyte count is an index that is relatively unaffected by inflammation and infection and reflects the immunological capacity over a certain period. Total cholesterol levels were not affected by dyslipidemia or statin use in this study and may reflect the patient’s nutritional status. Ignacio de Ulibarri et al. defined the categorization (as shown in **Table 1**) based on reported information and empirical findings and added double weight to the albumin score; however, they stated that this could be adjusted in the future.^8)^ In the future, an optimized CONUT score will be a better indicator for patients with CLTI.

This study had a number of limitations. First, it was carried out at a single center. Second, it was a retrospective study. Third, there was selection bias. The suboptimization of the scoring system also constituted a limitation. Thus, further research is needed that includes an endovascular treatment group. However, even in the current situation, patients with promising life expectancy should not easily avoid bypass surgery and choose endovascular treatment as their first choice simply because they have many comorbidities or are on dialysis. On the other hand, there are cases in which endovascular treatment is preferable over bypass surgery when high life expectancy is not expected, and primary major amputation may be an option when the limb salvage rate is also expected to be low. In this study, specifically in the low CONUT score group (0–2 points), both survival and limb salvage rates were expected to be good; therefore, active bypass surgery is an option. On the contrary, in the high CONUT score group (8–12 points), both survival and limb salvage rates were expected to be poor; therefore, minimally invasive endovascular treatment, primary major amputation, or even non-surgical treatment may be an option instead of bypass surgery. In the intermediate CONUT score group (3–7 points), the life expectancy was expected to be poor even if limb salvage was expected. Therefore, the choice of bypass surgery, endovascular treatment, or primary major amputation should be considered after further risk stratification. Based on this study, life expectancy-predicting factors in each treatment group should be sought and optimized, and by combining these indicators, we may be able to provide more appropriate individualized treatment to patients with CLTI.

In patients with CLTI, even when the CONUT score is found to be high, there is often no time for implementing adequate nutritional and immunological interventions before surgery. Furthermore, in these patients, infections caused by ulcers and necrotic lesions may be responsible for low serum albumin levels, and ischemic pain may be indirectly related to a decrease in oral intake. It is possible that infection control and adequate pain control by appropriate preoperative drainage may indirectly lead to the rapid correction of the nutritional and immune statuses.

## Conclusions

For CLTI, the CONUT score can serve as an objective predictive indicator of survival and limb salvage in patients undergoing surgical bypass and may be useful in selecting the optimal treatment.

## Notes

This is a translation of J Jpn Coll Angiol 2020; 60: 35–41.
